# Gleanings in Science and Medicine

**Published:** 1893-05-06

**Authors:** 


### Gleanings in Science and Medicine.

Berlin boasts of its four lady doctors already—whether the profession will be further increased through feminine assailants remains to be seen; but if Herr Baumbach's motion is carried, which he brought forward lately with much eloquence in the Reichstag, asking that women be permitted to present themselves for the Staat's Examen—with a view to practising medicine—if this is finally carried we can only imagine that the existing female practitioners will have their numbers largely augmented in Berlin as in other German towns.

A correspondent to the *Lahore Civil and Military Gazette* gives an account of his personal experience with regard to the protective effect of certain colours against the sun's rays. No one, the writer observes, has ever been a victim to sunstroke or sun fever through a dark source of heat, or even to one which, like the furnaces in the Arsenal, is not possessed of any great degree of chemical energy; for it is not the heat rays which thus act injuriously, but the chemical ones. The writer continues, that as the photographer treats his plates by enveloping them in yellow or red, so he treated his body. All the linings of his hats and coats were yellow, with the satisfactory result that after a trial of five years of this plan, even often under circumstances of extreme exposure, there was no return either of fever or sunstroke, to both of which the writer declares himself to have previously been a victim, but on the two occasions when he omitted this precaution a recurrence of the fever did take place.

This is pre-eminently an age of advertisements. Whether the next century will witness a further elaboration of the same plan remains yet to be seen. A society has lately been formed heading itself under the hopeful title of "The National Society for Checking the Abuses of Public Advertising." But beyond the actual pain to our æsthetic sensibilities caused by this pernicious habit, which the reforming world would abolish, the greater harm by far is to the advertisers themselves, who are their own worst enemies, for few firms can support the enormous drain on their funds, through this immense outlay, which out-advertising one another entails. It stands to reason that if A's boots are good, it is compulsory for B to declare his better, and so on down the alphabet, under the advertizing of Z, finding all known medium of attracting public attention already appropriated, has to turn from the known to the unknown and discover some blatant new means of outrivalling his co-manufacturers. The best friends which these great advertisers possess are those who now rise up in enmity for their suppression, and call themselves members of the longest named society extant; for the actual "returns" of this "En Evidence" system, are not always in proportion to the outlay, and more than one company has ruined itself through its advertisements. America, of all countries, bears the greatest testimony to the fact that nothing is held sacred from this system; the very beasts in the field bear the mark of the desecrating hand; it is not uncommon to witness a herd of oxen irresponsively but gaudily recommending on their one side Anti-Gout Pills, and on their other Madame Zara's Bonnets.

